# Climate smart agriculture, farm household typologies and food security: An *ex-ante* assessment from Eastern India

**DOI:** 10.1016/j.agsy.2017.09.007

**Published:** 2018-01

**Authors:** Santiago Lopez-Ridaura, Romain Frelat, Mark T. van Wijk, Diego Valbuena, Timothy J. Krupnik, M.L. Jat

**Affiliations:** a International Maize and Wheat Improvement Center (CIMMYT), Sustainable Intensification Program and CGIAR Research Program on Climate Change, Agriculture and Food Security (CCAFS), Apdo, 6-641 06600, México, D.F., Mexico; b International Livestock Research Institute (ILRI), Livestock Systems and the Environment, P.O. Box 30709, Nairobi 00100, Kenya; c International Center for Tropical Agriculture (CIAT), Sub-regional Office for Central America Planes de Altamira, de Pizza Hut Villa Fontana 1 cuadra al oeste Edificio CAR III, 4to. Piso Apartado, LM-172 Managua, Nicaragua; d International Maize and Wheat Improvement Center (CIMMYT), Sustainable Intensification Program and MAIZE CGIAR Research Program, House10/B, Road 53, Gulshan-2, Dhaka 1213, Bangladesh; e International Maize and Wheat Improvement Center (CIMMYT), Sustainable Intensification Program and CGIAR Research Program on Climate Change, Agriculture and Food Security (CCAFS), NASC Complex, DPS Marg, New Delhi 110012. India

**Keywords:** Socio-ecological system, Scenario evaluation, Climate change, Conservation agriculture, Livestock, Drought, Bihar

## Abstract

One of the great challenges in agricultural development and sustainable intensification is the assurance of social equity in food security oriented interventions. Development practitioners, researchers, and policy makers alike could benefit from prior insight into what interventions or environmental shocks might differentially affect farmers' food security status, in order to move towards more informed and equitable development. We examined the food security status and livelihood activities of 269 smallholder farm households (HHs) in Bihar, India. Proceeding with a four-step analysis, we first applied a multivariate statistical methodology to differentiate five primary farming system types. We next applied an indicator of food security in the form of HH potential food availability (PFA), and examined the contribution of crop, livestock, and on- and off-farm income generation to PFA within each farm HH type. Lastly, we applied scenario analysis to examine the potential impact of the adoption of ‘climate smart’ agricultural (CSA) practices in the form of conservation agriculture (CA) and improved livestock husbandry, and environmental shocks on HH PFA. Our results indicate that compared to livestock interventions, CA may hold considerable potential to boost HH PFA, though primarily for wealthier and medium-scale cereal farmers. These farm HH types were however considerably more vulnerable to food insecurity risks resulting from simulated drought, while part-time farmers and resource-poor agricultural laborers generating income from off-farm pursuits were comparatively less vulnerable, due in part to their more diversified income sources and potential to migrate in search of work. Our results underscore the importance of prior planning for development initiatives aimed at increasing smallholder food security while maintaining social equity, while providing a robust methodology to vet the implications of agricultural interventions on an *ex ante* basis.

## Highlights

• Five primary farming system types were identified in Bihar, India.
• Conservation agriculture boosts food security for wealthy and medium-scale farmers.
• Simulated drought has considerable negative impacts on all farm types.
• Farmers with diversified income are however less vulnerable to environmental shocks.
• This simple *ex-ante* approach may benefit agricultural development planners.

## 1 Introduction

The global diversity of smallholder farming systems and associated livelihood strategies reflects the intrinsic interaction of social-ecological processes and factors at different organizational levels. Proper characterization of this diversity is therefore an important step towards delineating the appropriate social-ecological niche for different technological and policy options (Descheemaeker et al., 2016, Ojiem et al., 2006). When combined with geographic analysis, recommendation domains for agronomic technologies, management practices, and farming systems can be developed, with the ultimate goal of increasing the efficiency of development efforts by accelerating smallholder farmers' adaptation and adoption of productivity increasing technology products (Sumberg and Reese, 2004).

In intensive cereal based farming systems, the successful development of resource use efficient management practices requires coherence with farmers' resource endowments, ability and interest to invest in diversified crop and livestock species, crop and livestock management techniques and livelihood options, as well as with the full range of activities carried out by farming households. Grouping farming systems in terms of their resources and livelihood activities, as well as agricultural management practices, is now common. Farming system typologies have been used for nearly two decades to capture the diversity of farming systems (Landais, 1998), and are increasingly used to provide guidelines for the development of agricultural innovations and to better understand their implications for climate change (Berre et al., 2016, Chopin et al., 2015, Douxchamps et al., 2015, Kuivanen et al., 2016, Pacini et al., 2014, Tittonell, 2014).

Foresight of the possible impact of climatic shocks and technological alternatives is also an indispensable step towards the delimitation of appropriate recommendation domains for ‘climate smart’ agriculture (CSA). CSA aims to simultaneously increase agricultural productivity, food security, and farmers' adaptive capacity to climate extremes, while also lowering greenhouse gas emissions (Campbell et al., 2014). *Ex-ante* foresight can also aid in the design of technological alternatives (and accompanying delivery pathways and policies) for CSA, and development interventions intended to improve the smallholder livelihoods (cf. Rosenstock et al., 2014). The complexity and diversity of farm household livelihood strategies however necessitates careful focus on key indicators that reflect changes in vulnerability or resilience, with particular emphasis on food security (Frelat et al., 2016).

In South Asia's intensively cropped Indo-Gangetic Plains (IGP), an estimated 640 million people live in extreme poverty and rely on cereals for primary subsistence (Saharawat et al., 2010). The IGP encompasses the Ganges, Indus, and Brahmaputra river basins where rice, wheat and maize are the most commonly rotated cereals. Rice-wheat rotations in particular predominate on > 13 million ha (Chauhan et al., 2012, Jat et al., 2016). The IGP nonetheless has a high degree of spatial variability in terms of poverty, with a clear low-to-high gradient of food insecurity moving from west-to-east (Erenstein and Thorpe, 2011). Farmers tend to have larger herds and farm sizes, more access to irrigation, and higher cropping intensity in the west, all of which influences household food security (Erenstein et al., 2010, Erenstein and Thorpe, 2011). Yield gaps however remain common in the IGP, ranging from 14 to 47%, 18–70%, and 36–77% for wheat, rice, and maize, respectively. These gaps widen in the eastern IGP, broadly correlating with the region's increased poverty, farmers' low investment capability and aversion to risk, and increasing in energy and input costs, in addition to climactic variability (Aravindakshan et al., 2015, Jat et al., 2016). Pulses, oilseeds, and mixed crop-livestock systems are also common, as is farmer engagement in seasonal and semi-permanent migration and off-farm labor (Erenstein and Thorpe, 2011).

Farmers in the IGP are also vulnerable to climate change (Jat et al., 2016, Sapkota et al., 2015). Increasing temperatures reduce the winter season wheat crop's duration which, when combined with terminal heat stress and drought, can substantially lower productivity (Arshad et al., 2016, Krupnik et al., 2015a, Krupnik et al., 2015b). Eastern India's Bihar State has been identified as one of most vulnerable regions to climate change due to heat, drought and flood risks, in addition to increasingly erratic monsoon precipitation (Sehgal et al., 2013, Chhabra and Haris, 2015). As India's third most populous state, over 90% of Bihar's inhabitants live in rural areas. 81% depend on agriculture, although food insecurity remains common (Krishna and Kumar, 2014). Development planners nonetheless hope to convert Bihar to India's ‘future food bowl’ by dramatically boosting cereals and livestock production (Singh et al., 2009, Laik et al., 2014). This is a formidable challenge given the state's generally unfavorable biophysical and climactic environment, high degree of farm fragmentation, inadequate infrastructure, and weak institutions and markets (Laik et al., 2014).

Over the last decade, alternative cropping systems employing the principles of CSA have been have been developed in the form of conservation agriculture through on-station and on-farm validation trials across Bihar (Jat et al., 2014, Laik et al., 2014, Sapkota et al., 2015). These innovations include alternatives to intensive tillage for rice establishment that mitigate global warming potential, alongside rotational options for direct seeded maize and wheat establishment with the retention of crop resides as a surface mulch to conserve soil moisture (Singh et al., 2012, Jat et al., 2014, Laik et al., 2014). When carefully implemented, these practices can reduce production costs, energy demand, and greenhouse gas emissions, while also maintaining or augmenting yield (Gathala et al., 2013, Jat et al., 2014, Jat et al., 2016, Gathala et al., 2016). These outcomes qualify these practices under the rubric of CSA (Sapkota et al., 2015). Not all technologies are however likely to generate equal income or food security benefits for all smallholder households. For example, milk production comprises an important source of nutrition and income generation for some farmers' livelihood systems in eastern India (Erenstein et al., 2010, Erenstein and Thorpe, 2011) and therefore, increases in cereal crop productivity may have less impact on their food security or income. The poor fit of many widely-promoted agronomic technologies has been corroborated by their low and differentiated adoption (Erenstein and Laxmi, 2008, Singh et al., 2012), further challenging the goal of increasing Bihar's cereal productivity.

In this paper, we demonstrate how the use of farming systems typologies and an innovative food security model can be used to explore and assess the impact of CSA practices, improved animal husbandry, and climactic shocks on the food security of farm households on an *ex-ante* basis. Using survey data from 269 farmers in six villages and three districts of Bihar, we apply a multivariate statistical methodology for typology construction combined with the calculation a simple yet robust food security indicator. We follow with an analysis of different agronomic intervention and climactic risk scenario analyses, with interpretation of the results differentiated by predominant farming system type. We conclude by discussing the ways in which this methodology can be used to generate insight into the advantages and constraints of alternative agricultural interventions and scenarios, in order to better target interventions for more equitable development among smallholder farmers while reducing their vulnerability to climate change related risks.

## 2 Methodology

### 2.1 Study location and survey details

**Fig. 1:**
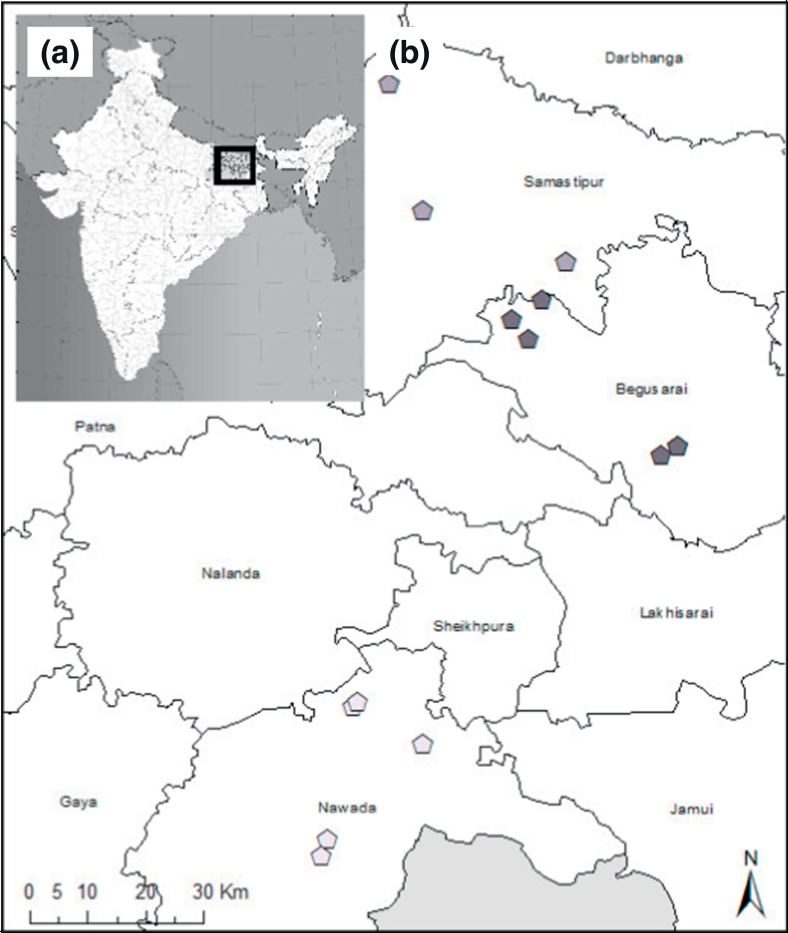
Map (a) South Asia with (b) detail of Bihar and the surveyed 18 villages. Point data indicate each village in different districts. Nawada (light grey) is South of the Ganges, Begusarai (dark grey) is the Ganges North ridge, and Samastipur (intermediate grey) is in the North fertile plain. Some villages may overlap due to map scale.

Administered in 2010–2011, the Cereal Systems Initiative for South Asia (CSISA) farm household (HH) survey catalogued farming systems and livelihood pursuits in the IGP (Pede et al., 2013). The survey included intensive sampling in Bihar, selected because of its dependency on agriculture for food security, and because of its ranking as India's poorest state (RBI, 2013). Bihar also hosts a number of long-term agronomic experimental platforms that compare conventional crop management with CSA practices, thereby providing data for simulation analyses (see Section 2.4). Surveys were administered in Bihar's Begusarai, Nawada and Samastipur districts. Within each, three administrative blocks were chosen, after which two villages per block with 18 HHs each were selected (Fig. 1). Each layer of this selection process was randomized, resulting in a dataset of 269 HHs. The survey instrument was organized into five sections, including (i) general farm and HH characteristics, including land use and capital, (ii) farm input and labor use, (iii) experience with and adoption of field crop and horticultural production technologies and practices, (iv) livestock production, with emphasis on dairy, and crop residue management (including use as animal feed), and (v) off-farm HH income sources and financial expenditures.

### 2.2 Typology construction

#### 2.2.1 Selection of variables

We explored the diversity of Bihar's farming systems using typological analysis (Berre et al., 2016, Cortez-Arriola et al., 2015, Pacini et al., 2014, Tittonell, 2014). Typologies can be developed using structural (farm assets and resources) or functional (livelihood pursuits) variables, or both (Tittonell, 2014). We selected and computed variables representing structural and functional features of farming systems, the latter related mainly to farmers' primary crop and livestock systems. Thirty-two variables were computed in total (Table 1). The remaining variables were related to farm mechanization, for which we employed a custom scoring system by aggregating information about the use and ownership of irrigation pumps, tractors, threshers, fodder choppers, and pesticide sprayers. We firstly considered the category of use for each of these machines, and accounted for the relative importance of each item by assigning scores of 2, 1, and 0 if they were owned, rented, or owned but not used, respectively. This is important for overcoming any bias that may result from items owned but not used, and by indicating if the machine is owned (which permits flexible use) or rented (which is less flexible in use and employed only for specific tasks). Each item's relative importance for farm intensification was then tabulated (i.e. a tractor use is comparatively more consistently important than sprayer use as land preparation is prerequisite to cropping). After assessing each HH's frequency of ownership or rental, machinery types were ranked in descending order of frequency, assigning a low score to popular items that aid in but are arguably not essential to crop production (e.g. pumps), and high scores to low-frequency items that are prerequisite to establishing a crop (e.g. tractors). Weights were then applied to the following types of farm equipment: 2 for pumps and threshers, 3 for fodder choppers, 4 for sprayers, and 5 for tractors. These weights were then modified by subtracting the value of one if the items were rented instead of owned (e.g. tractor rental is equivalent to 5 − 1 = 4, and is thus valued higher than pump ownership with a score of 2).

**Table 1:** Structural and functional variables employed for the construction of household typologies.

Variable	Unit
Household land and workforce	
Total land managed	Hectares
Rented land	Percent^a^
Cultivated land (summer *kharif* season)	Percent
Cultivated land (winter *rabi* season)	Percent
Cultivated land under irrigation	Percent
Household workforce involved in farming	Number of people
Household workforce engaging in off-farm employment	Number of people
Percentage of total workforce dedicated to farming	Percent
Household size to managed land ratio	HH members ha^− 1^
Livestock assets and density	
Tropical livestock unit (TLU) density	bTLU ha^− 1^
TLU number	Number of TLUs
Market integration and household income	
Harvested crops sold	Percent
Income from off-farm sources	Percent
Income from livestock	Percent
Income from crop production	Percent
Cropping patterns	
Land dedicated to wheat (winter *rabi* season)	Percent
Land dedicated to rice (summer *kharif* season)	Percent
Land dedicated to maize in (winter *rabi* season)	Percent
Land dedicated to maize in (summer *kharif* season)	Percent
Land dedicated to fodder	Percent
Land dedicated to legumes	Percent
Land dedicated to vegetables	Percent
Land dedicated to oilseeds	Percent
Land dedicated to industrial crops	Percent
Crop diversity	Crop number farm^− 1^ year^− 1^
Different crops grown during the summer *kharif* season	Crop number farm^− 1^ in *kharif*
Crop residue allocation	
Wheat and rice residues used for fodder	Percent
Wheat and rice residues sold	Percent
Wheat and rice residues burnt in the field	Percent
Wheat and rice residues used as fuel	Percent
Wheat and rice residues left in the field	Percent
Farm mechanization	
Mechanization score	Custom score^c^

a Percent of total land, crops, cropping patterns, or crop residues.

b TLU indicates tropical livestock units (HarvestChoice, 2011).

c Detailed in Section 2.2.1.

#### 2.2.2 Data clustering

Three steps were taken to build our HH typology. The first step reduced the dimensionality of the data and identified primary patterns and variability by applying principal component analysis (PCA) using R (R 3.1.1. Core Team, 2014) with the package ade4 (Dray and Dufour, 2007). Selection of the relevant principal components (PCs) was performed by scree test (Cattell, 1966). In the second step, we employed hierarchical clustering analysis on the new orthogonal data projection made by the selected PCs. Cluster numbers were determined in the last step, after which we constructed a dendrogram of an ascendant hierarchical classification performed using Ward's criterion (Ward, 1963). We enforced a decision rule set regarding where to cut dendrogram branches by searching for the maximum average silhouette width (measures derived from the comparison of intra-class similarity, with high and low inter-class similarity separated) of different *k*-means clustering solutions with varying cluster numbers (Rousseeuw, 1987).

### 2.3 Food security assessment

**Fig. 2:**
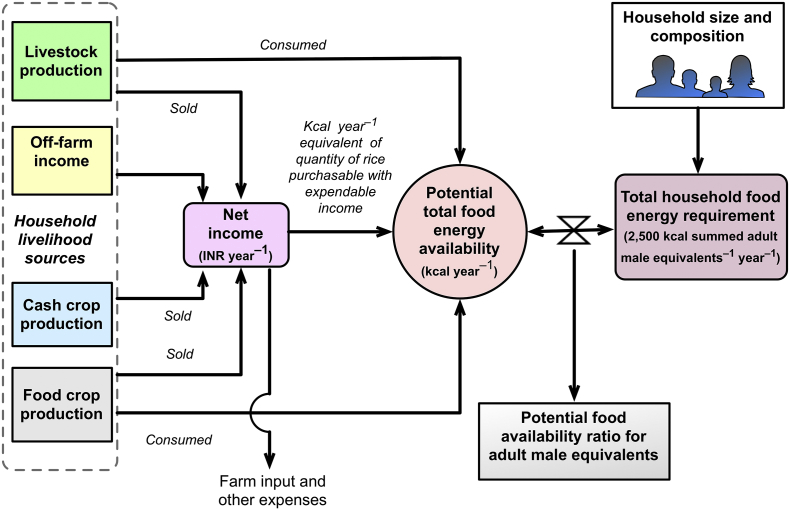
A simple model of the potential food availability ratio expressed in energy equivalents, showing direct and indirect forms of food availability generation and consideration of household required caloric availability (adapted from Frelat et al., 2016), using a minimum daily threshold of 2500 kcal person^− 1^ (in adult male equivalents) for all days of the year. If the ratio is > 1, the household is considered “food secure”. If < 1, the household is “food insecure”.

There are literally hundreds of definitions of food security (FAO, 2003). The term usually implies that people (at whatever scales considered, from households to nations to regions) have equal and sustained physical and economic access to a sufficient amount of safe and nutritious food to meet daily caloric requirements and to maintain an active and healthy lifestyle (cf. FAO, 1996). While comprehensive, the complexity of this definition makes quantification difficult. Food availability is conversely the basis for food access and food security (Mainuddin and Kirby, 2015), especially at the household level. Given our available data, we therefore adapted a simple measure of potential HH food availability ratio as an indicator of food security, based on Frelat et al. (2016) (Fig. 2).

This indicator quantifies potential food availability (PFA) as an index, calculated on a kilocalorie basis, per individual farm HH based on reported production and consumption of crop and livestock products, as well as consumption of food purchased from money earned through off-farm employment and/or sales of produced crop and livestock products. Although it does not measure actual consumption, this simple indicator is strongly correlated with other indicators of food security and nutrition. Examples include the household level diet diversity score and the hunger and food insecurity access scale, although PFA is relatively more straightforward to measure (Hammond et al., 2016). For this reason, it has been widely used to assess the drivers of food security in Sub-Saharan Africa (Frelat et al., 2016, Ritzema et al., 2017), although it has not yet been applied anywhere in South Asia.

The PFA of an observed HH is a function of their direct and indirect food consumption, which we represent in energy equivalents (kcal). Direct consumption of food and livestock products refers to a given HHs' use of products produced on their farm converted into energy equivalents, as determined by Eq. (1):

(1) $${\mathrm{PFA}}_{\text{direct energy}}=\sum_{\mathrm{fc}}Y_{\mathrm{fc}}\times E_{\mathrm{fc}}\times \theta_{\mathrm{fc}}+\sum_{l}Y_{l}\times E_{l}\times \theta_{l}\;$$

where *Y*_*fc*_ is the yield of each food crop *fc* (kg farm HH^− 1^), *E*_*fc*_ is the energy density of each crop and crop constituent (kcal kg^− 1^), and *θ*_*fc*_ is the fraction of each crop consumed by the HH. *Y*_*l*_, *E*_*l*_ and *θ*_*l*_ refer to the livestock equivalents of the same variables, respectively. The primary livestock product was milk (l cow^− 1^ day^− 1^). Energetic coefficients for crop and livestock products were determined from USDA (2015) coefficients.

HHs may also increase food availability through indirect consumption using cash reserves derived from cash income (*C*_*INR*_, Indian Rupees (INR)) earned through sales of farm products as well as off-farm income sources, after subtracting expendable income reinvested in farm inputs, as described in Eq. (2):

(2) $$C_{\mathrm{INR}}=\sum_{\mathrm{fcs}}Y_{\mathrm{fc}}\times \beta_{\mathrm{fc}}\times \left(1-\theta_{\mathrm{fc}}\right)+\sum_{\mathrm{ls}}Y_{l}\times \beta_{l}\times \left(1-\theta_{l}\right)+\sum_{\mathrm{of}}\beta_{\mathrm{of}}-\sum_{\mathrm{ie}}\beta_{\mathrm{ie}}\;$$

where *β*_*ie*_ denotes farm input expenses (Indian Rupees (INR) unit^− 1^), for example machinery rental, seeds, fertilizers, irrigation, transportation, or labor. The addition of the *s* subscript to *fc* and *l* indicate food crops and livestock products sold to generate cash income. *β*_*fcs*_ and *β*_*Lls*_ each represent the median market price (INR kg^− 1^) at which food crops and livestock products were sold, as reported by survey respondents. Cash crops were defined as crops of which > 90% of the annual produce is sold (i.e. *θ*_*fc*_ < 0.1). Crops like cotton were therefore treated as cash crops, while some food crops, for example maize, may be included as a cash crop if the farm HH sells at least 90% of annual production. *β*_*of*_ stands for income generated through off-farm employment, investments, or remittances, derived from farmers' reported percentage of total HH income.

In order to standardize the values resulting from indirect consumption calculations, indirect food availability data were converted to the energetic equivalent (*E*_*rice*_,kcal kg^− 1^) of the amount of the primary standard staple food in the study area, in this case milled rice (*Oryza sativa*), which could be purchased with earned income (*C*_*INR*_) at the median reported market price in each district (*P*_*rice*_). This is a simple way of transforming cash into energy and is an estimation of the maximum level of energy that a household can obtain from reported cash generation through sales and off farm income (Frelat et al., 2016, Hammond et al., 2016):

(3) $${\mathrm{PFA}}_{\text{Indirect}}=C_{\mathrm{INR}}\times \frac{E_{\mathrm{rice}}}{P_{\mathrm{rice}}}$$

In our calculations, we accounted only for food crops, livestock products, or cash crops consumed or sold. Products lost due to spoilage or wastage were not included. The total amount of food potentially available for the farm HH (kcal HH^− 1^) was then calculated:

(4) $${\mathrm{PFA}}_{\text{total}}=\;{\mathrm{PFA}}_{\text{direct energy}}+{\mathrm{PFA}}_{\text{indirect energy}}$$

In order to compare the potential food available (in kcal) at the HH level, we first disaggregated HH members by gender and age classes as advised by the FAO (2001), in order to estimate adult male equivalents (also referred to as capita below), based on energy requirements for members of three age classes. To obtain capita equivalents, we attributed a weight of 0.4 to the number of children between zero and six, 0.7 for children between six and 15 years old, one for males older than 15, and 0.9 for women older than 15. Total daily farm HH energy requirements (*E*_*hh*_) were then computed:

(5) $$E_{\mathrm{hh}}=\;\sum_{i}n_{i}\times \alpha_{i}$$

where *n*_*i*_ is the number of individuals in each age class *i*, and *α*_*i*_ is the energy requirement (kcal person^− 1^ day^− 1^) in each age class, based on the standard per capita equivalent of 2500 kcal day^− 1^. The above information were then integrated to provide potential food availability for one year:

(6) $${\mathrm{PFA}}_{\text{ratio}}=\;\frac{{\mathrm{PFA}}_{\text{total}}}{\;365\;\;\text{days}\;{\text{year}}^{–1}\times E_{\mathrm{hh}}}$$

### 2.4 Scenario analysis

We considered three scenarios to simulate the effect of technology adoption or crop failure, in order to quantify their hypothetical impact on the PFA ratio of households (Table 2). These scenarios model a simplified version of possible changes in agricultural production, and are intended as a heuristic *ex ante* tool to judge the potential impact of development interventions or risks on HH food availability.

**Table 2:** Summary of three scenarios applied to the farm household food security model to simulate the potential effect of select agronomic and livestock production technology adoption, and of climactic shocks in the form of drought.

Scenario		Median observed effect	
		Food production benefit	Production costs
1	Intensive rice-wheat rotations utilizing conservation agriculture^a^	• Rice yield = 6.3 t ha^− 1^ • Wheat yield = 5.8 t ha^− 1^ • Rice-wheat system yield = 12.1 t ha^− 1^	• Rice = 27,500 INR (USD 410) ha^− 1^ • Wheat = 24,750 INR (USD 369) ha^− 1^ • Rice-wheat system = 52,250 INR (USD 779) ha^− 1^
2	Increased livestock production	Increase of milk production by 50%^b^	No direct economic costs; rather indirect costs in the form of foregone forage sales are included as crop residues are redirected to farm livestock.
3	Climactic shock: Catastrophic drought	No benefit	90% decrease in rice, wheat, millet, maize, sorghum, and oat production

a Following Jat et al. (2014).

b Note that in our dataset, relatively little or no additional (i.e. purchased) inputs were found for households that have higher milk production than those with lower milk production, irrespective of livestock number. Rather, increased milk production appears to be the result of relatively simple changes in livestock husbandry and improved management practices, with emphasis on increasing the volume of feed directed to lactating animals in the form of crop residues.

#### 2.4.1 Scenario 1: conservation agriculture

The first scenario considers the adoption of ‘climate smart’ agricultural practices frequently promoted to intensify rice–wheat rotations while limiting negative environmental externalities by using conservation agriculture principles (Aravindakshan et al., 2015). Using long-term experimental data from Bihar's rice-wheat systems, we simulated the effect of farmers achieving rice and wheat yields and associated gross margins of the most agronomically and economically efficient treatment reported by Jat et al. (2014). The experimental cropping pattern included wheat established using zero-tillage rotated with directly sown dry seeded rice, also under zero tillage, with 50% and 25% of rice and wheat residues, respectively, retained as mulch. Starting from initial establishment in 2006–2007, with baseline rice-wheat yields of 6.43 t ha^− 1^, the linear slope of the annual rice-wheat system yield increase for this treatment was 0.9 t ha^− 1^ (*R*^2^ = 0.88), These practices also generated an annual increase of INR 18,032 (USD 267) ha^− 1^ (*R*^2^ = 0.92) from the baseline year, which were simulated as additional cash income from rice and wheat sold, while reducing diesel costs and hence greenhouse gas emissions from repetitive tillage, as reported by Jat et al. (2014). Yields and profits from the last year of the experiment were applied as detailed in Table 2. These practices also required increased seed and herbicide costs, which were accounted for in our calculations. All other farm HH parameters were left unchanged.

#### 2.4.2 Scenario 2: increased livestock productivity

The second scenario explores the potential impact of productivity enhancing livestock management practices on the PFA ratio. Such practices can include improving the quality of feed, disease prevention and management, as well as use of improved breeds, among others. Because no experimental data were available to simulate productivity enhancing management practices for livestock on Bihar, we followed methods employed by Ritzema et al. (2017) to assess the impact of livestock production intensification on HH food security. The impact of an assumed 50% increase in the production of milk, which could be obtained through a combination of the management practices mentioned above, was examined with all other farm parameters left unchanged.

#### 2.4.3 Scenario 3: climactic shock

In the final scenario, we assessed the vulnerability of Bihar's farming systems and food security to climatic shock in the form of drought. Our purpose was to examine the effect of an extreme environmental shock on the behavior of the model and resulting data, and to examine what the differential effect of an extreme and severe drought event might be on each household type's food availability status. Drought and delayed onset of monsoonal rains have been observed in five of the last six years in Bihar, affecting 38 of the state's districts with variable yet significant crop losses (Kishore et al., 2014). We therefore examined the effect of drought by simulating an extreme 90% decrease in the production of key cereals grown by farm HHs, including rice, wheat (*Triticum aestivum*), maize (*Zea mays*), millet (*Pennisetum glaucum*), sorghum (*Sorghum bicolor*), and oats (*Avena sativa*). All other parameters were held constant.

## 3 Results

### 3.1 Farming system typologies

**Fig. 3:**
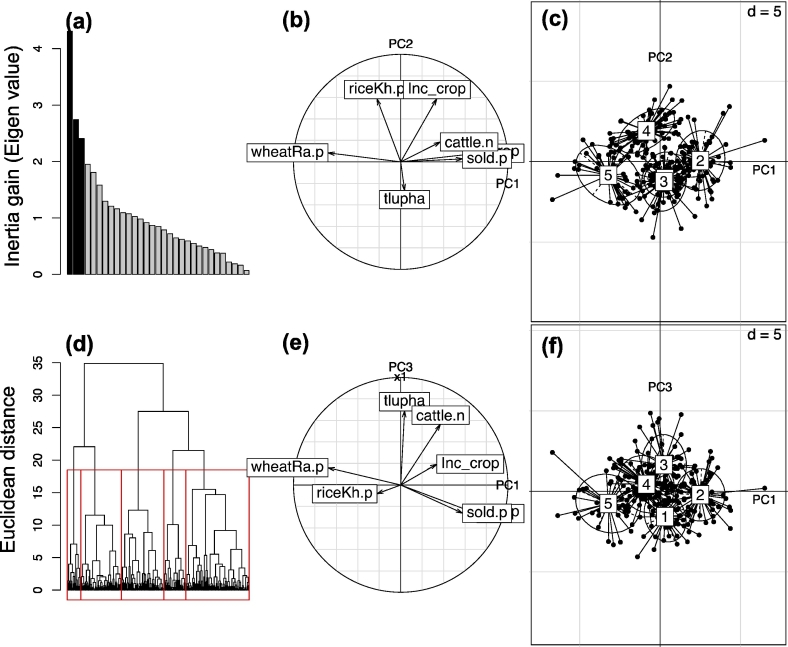
Results of the Bihar principle components and cluster analysis. (a) Variability explained by successive principle components expressed as inertia gain. The first three principle components explain 13%, 22% and 30% of the total cumulative variability. (b) Projection of variables on first two principle components, (c) Clusters projected on first two principle components. (d) Hierarchical cluster analysis dendogram depicting five clusters projected from inclusion of three principle components. (e) Variables projected on the first and third principle component. (f) Clusters projected on all three principle components.

Of the 32 variables measured in surveys, scree plots of the Eigen values resulting from the PCA indicated that the diversity in farm household characteristics was associated with three principal components (PC), together explaining 30% of the variability (Fig. 3A). These variables were related to the diversity and intensity of cropping activities, market integration, the relative importance of farming in income generation, as well as herd size and stocking rate (Fig. 3B). The first two PCs spread out farm households in terms of the number of crops grown, the percentage of land dedicated to wheat in the dry season, the percentage of land dedicated to rice, and the proportion and importance of crop products sold for income generation. Combined, these two principle components explain 22% of variance. The third PC is related to the level of livestock resources and stocking rate (Fig. 3E). Taken with the first two PCs, the addition of the third PC explained 30% of variance. Hierarchical clustering analysis indicated five main types of farm HHs across the three districts examined. Projecting these clusters on the first two principle components, only four groups are observable (Fig. 3C), while the addition of the third principle component and subsequent re-projection clearly differentiated five clusters (Fig. 3D and F).

Summing HH farm types across districts, the first cluster discernable is a group of 46 farmers who can be described as taking part in agriculture only on a part-time basis (17% of the sample). This group has mid-level resource endowments in terms of land and livestock (medians of 1.1 ha and 1 head, respectively) as well as in terms of mechanization (median score 5.2). These farmers can however be differentiated because their primary income source is derived from off-farm activities. These *part-time farmers* diversify their production of staple maize, rice and wheat with vegetables and oilseeds. One-third of their crop products are sold to the market.

The second cluster of 63 HHs can be described as wealthy farmers, representing 23% of the sample. This group is characterized by large land and livestock holdings (medians of 2 ha and 2 heads, respectively), with the highest mechanization levels in our dataset (median 6.0). These households farm year-round and are highly market-oriented, with median proportions of income from crop and livestock activities recorded at 60 and 30%, respectively. These *wealthy farmers* produce a high diversity of crops including rice, wheat, maize, oilseeds and vegetables, of which half are on average directed to the market.

A group of 50 HHs representing 19% of the sample make up the third cluster, which can be considered as *small-scale crop and livestock farmers*. This group manages a comparatively small amount cropland (median of 0.6 ha), which is sometimes rented-in. They have a relatively high HH size to managed land ratio (median of 12 adult equivalents ha^− 1^), with low mechanization scores (median 3.6). These farmers conversely have high stocking rates (median of 2 heads on 0.4 ha), intensive use of crop residues as animal feed (75% as fodder), and the highest proportion of income generated from livestock product sales (median 27.5%). Their field crop systems are based on maize, rice, and wheat as staples, although sales of these crops also significantly contributes to income generation (median 40% of income originating from food crop production).

Medium-scale cereal crop farmers comprise the fourth cluster of 72 farm HHs (27% of the sample), with land and livestock holdings medians of 1.2 ha and 1 head, respectively, and mid-level mechanization scores (median 4.4). Their income is however primarily dependent on food and cash crops sold (median of 70%). These *medium-scale cereal crop farmers* also dedicated the largest proportion of their land to rice-wheat rotations. Although they integrate livestock. Milk produced is primarily for home consumption and does not represent a source of income for this group.

Lastly, the fifth and smallest group of 38 HHs can be described as *resource-poor agricultural laborers* (14% of the sample). As most of their income is generated off-farm, farm size was comparatively small (median of 0.3 ha), with the greatest HH size to managed land ratio (median of 28.2 adult equivalents ha^− 1^). This group has the lowest level of mechanization (median score 1.9). Their cropping systems are dedicated exclusively to rice-wheat rotations to produce staples for home consumption only. The overarching majority of this group's income is derived from off-employment, mainly as laborers on other farms. Livestock products were an insignificant source of energy for this group. Most crop residues were either sold or used for fuel.

**Fig. 4:**
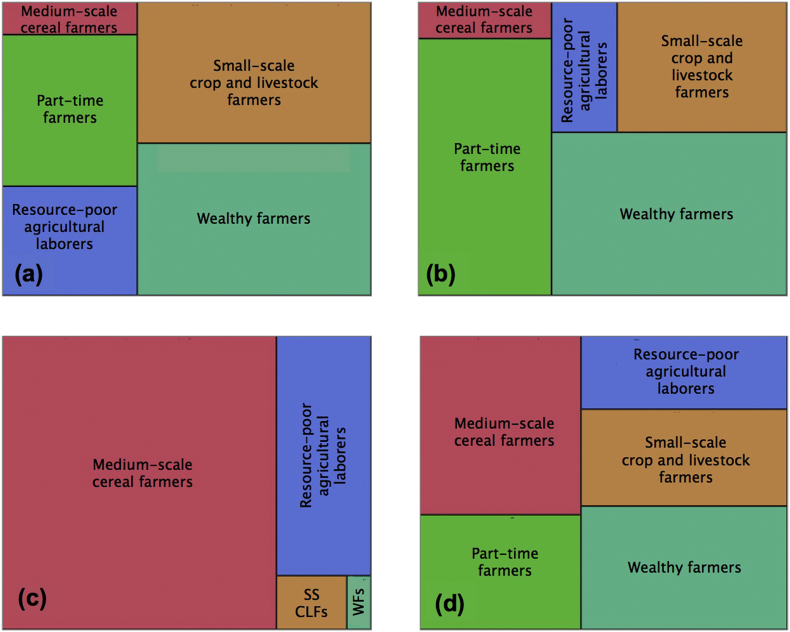
Hierarchical tree-structured maps depicting the proportions of the different farm household types in (a) Begusarai, (b) Samastipur, and (c) Nawada districts in Bihar and across all districts (d). SSCLFs indicates small-scale crop and livestock producers. WFs signifies wealthy farmers. *n* = 95, 88, and 86 farmers total in Begusarai, Samastipur, and Nawada, respectively.

All five clusters are observable in Begusari, Samastipur, although only four were found in Nawada (Fig. 4A–C). The former districts have similar farm HH type distribution with > 80% the observed HH belonging to either the part time farmer, wealthy farmer, or small-scale crop and livestock farmer groups. Nawada district conversely shows a different farm household type composition, with most HHs falling under the medium-scale cereal farming system group (cluster four). Summing farmers across districts, all five clusters were represented (Fig. 4D).

### 3.2 Potential food availability across clusters

**Fig. 5:**
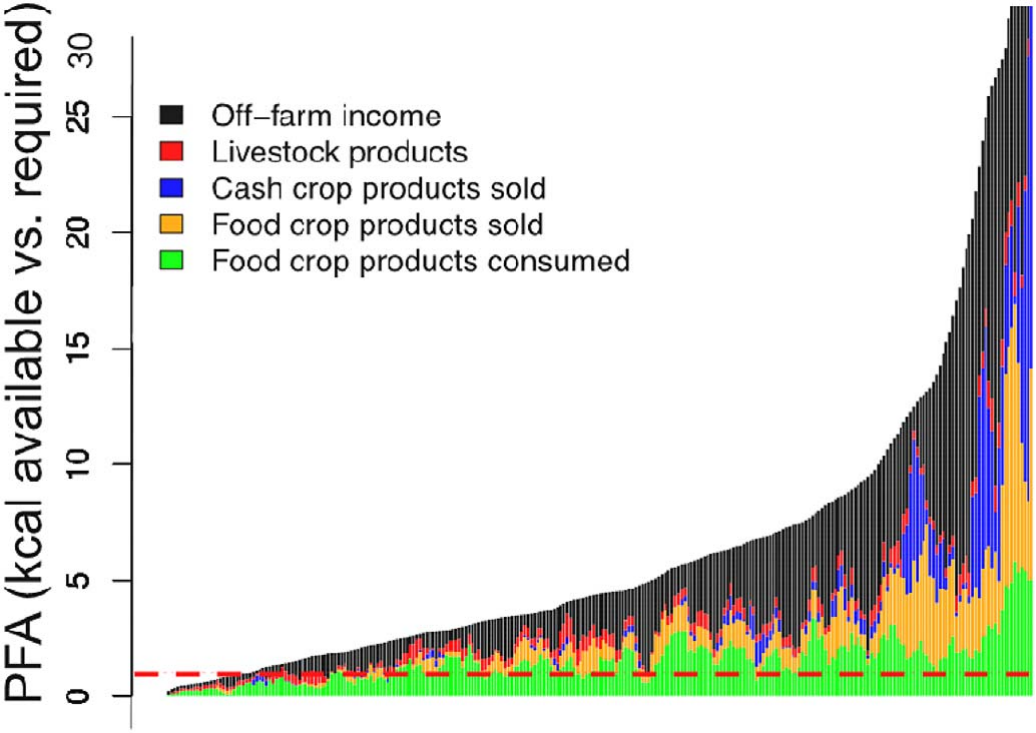
Distribution of potential food availability for households observed in Bihar. Farm households are ordered in ascendant order of their potential food availability ratio by moving average with window of five households. The dashed red line indicates a PFA of 1. Bin colors indicate livelihood strategies and potential sources of energy. (For interpretation of the references to color in this figure legend, the reader is referred to the web version of this article.)

Across all farm HHs and clusters, a large gradient in PFA was observed, with 10% of HHs unable to satisfy their basic caloric needs (Fig. 5). The contribution of food crop consumption to the PFA ratio decreases as HH food needs are satisfied, with progressively more HHs meeting food energy demand through cash crops, food crops sold, livestock, and off-farm income. Cash crops however contribute to the PFA ratio only for farm HHs with higher food availability ratios, suggesting that a minimum level of wealth and an ability to invest is prerequisite for cash crops to contribute to food security. Livestock is important for the maintenance of food security for poorer households (representing up to 20% of the PFA), but does not appear to be a crucial ingredient of Bihar's farming systems to increase PFA more generally. On the contrary, the significance of livestock to the PFA shows diminishing returns as PFA increases. Lastly, off-farm income plays a large role, with 40% of HH food resources purchased using off-farm income.

### 3.3 Cluster comparisons and potential food availability

**Fig. 6:**
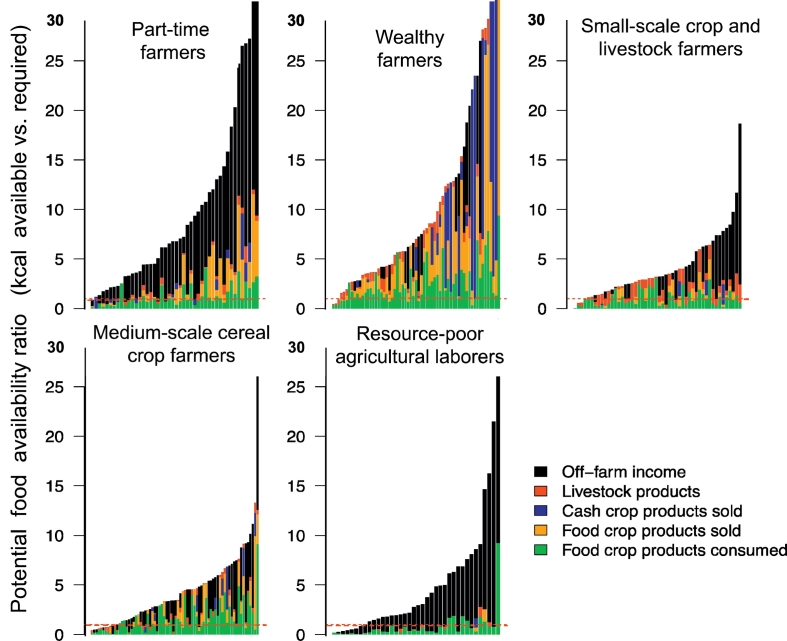
Distribution of potential food availability cluster grouping in Bihar. For details, refer to the legend of Fig. 5. (For interpretation of the references to color in this figure legend, the reader is referred to the web version of this article).

Examining food security in different farming system types, as expressed by the PFA ratios, considerable diversity in household livelihood strategies can be discerned (Fig. 6). Part-time farmers (cluster one) show considerable contribution of off-farm food sources (both purchased and directly consumed). Smaller farms appear to primarily consume their produce, while larger ones were more market integrated. All but one HH in this cluster were able to meet HH food energy requirements on an annual basis. In the second cluster, comprised of wealthy farmers, off-farm activities show little contribution to the PFA ratio. Their most important livelihood and hence potential sources of income are sales of both food and cash crops, with little livestock integration. These farmers are the most potentially food secure compared to others, with the steepest rate of increase in observed distributions, and 63% of HHs able to supply in excess of five-times the food energy required annually. This is indicative of an ability to hedge against food insecurity-related risks. Small-scale crop and livestock farmers make up the third cluster, with low rates of PFA ratio increase, and only 28% of HHs able to supply > 5 times the PFA ratio. While off-farm income makes a significant contribution to PFA for most HHs in this group, mixed crop-livestock systems contribute to the base of the PFA ratio. Off-farm income also becomes progressively more important for nine more food secure HHs on the right of the distribution, although maximum PFA ratio of this group (19) is lower than all others.

In the fourth cluster, medium-scale crops farmers, less homogeneity of livelihood activities were observed. While all livelihood activities contributing to the PFAs of different HHs, livestock is comparatively less uncommon. Eleven HHs in this cluster are food insecure. Finally, the fifth cluster of resource-poor agricultural laborers consists of HHs nearly entirely dependent on-off farm work to fulfill their food energy requirements, with some contribution of foods produced on their own farm. Despite being poor in on-farm resources, the majority of these farmers are nonetheless able to meet the minimum caloric threshold, with only eight HHs having PFA ratios < 1, although only 28% appear to be able to maintain PFA ratios > 5.

### 3.4 Scenario analysis

**Fig. 7:**
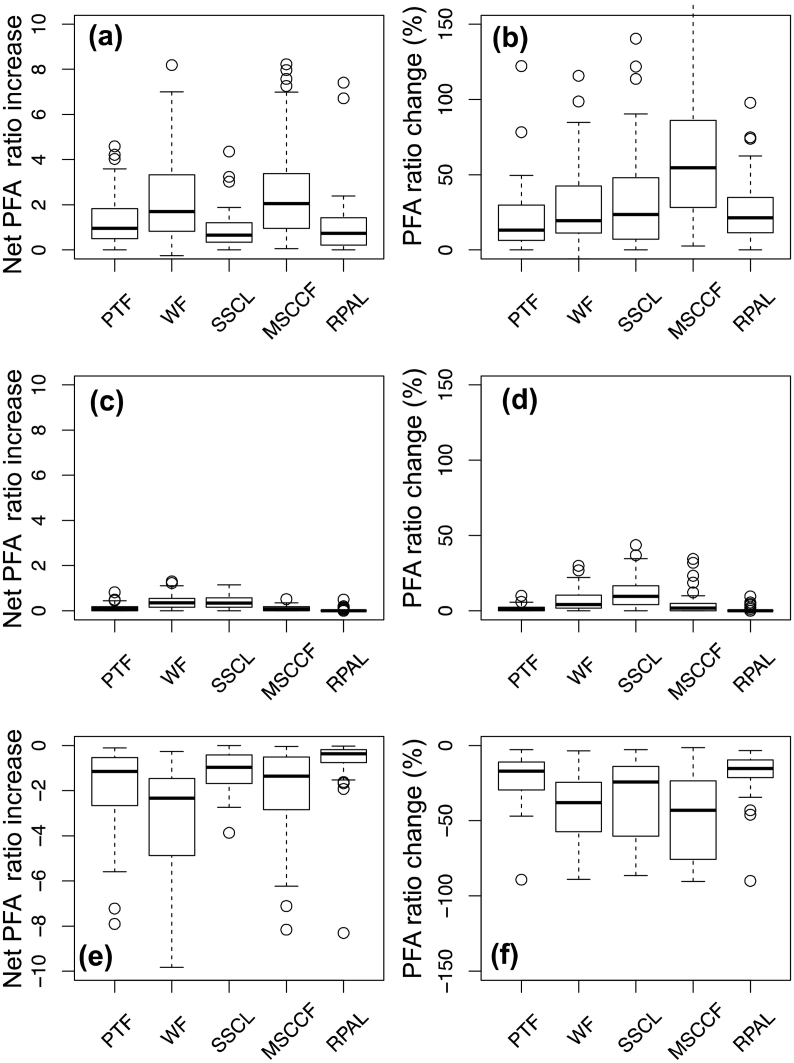
Box and whisker plot showing results of scenario analysis. (a) Net potential food availability (PFA) ratio increase and (b) percent change in PFA ratio from simulating the yield and productivity enhancing effects of conservation agriculture practices on rice-wheat cereal rotations (based on Jat et al., 2014). (c) Net PFA ratio increase and (d) corresponding percent PFA change for the 50% increase in daily milk yield scenario. (e) Net PFA ratio decrease and (f) percent PFA decline for the catastrophic drought scenario in which yields of all cereals were reduced by 90%. PTF = Part-time farmers, WF = Wealthy farmers, SSCL = Small-scale cereal and livestock farmers, MSCCF = medium-scale cereal crop farmers, and RPAL = Resource-poor farm laborers. Bold horizontal centerlines depict the median value. Upper and lower box ranges correspond to the upper 75th and lower 25th quartiles, respectively. Minimum and maximum values are depicted by the whiskers.

Scenario 1 considered farmers' use of zero tillage in intensive rice-wheat rotations with crop residue retention as mulch, as part of conservation agricultural practices. The scenario considered the replacement of farmers' rice and wheat land area with these practices, using the associated yield and profitability levels reported by Jat et al. (2014) to evaluate how PFA responded by farm HH type. Simulated adoption of these practices had a positive impact on PFA (Fig. 7A), most notably for medium-scale cereal crop farmers whose PFA ratios increased by a median 81% (Fig. 7B). This group tended to have large land holdings (median 1.2 ha) and a large reliance on field crop production (Fig. 6). Medium-scale cereal crop farmers appear to have benefited disproportionally due to their relatively low baseline median production values for rice (0.7 t ha^− 1^) and wheat (0.6 t ha^− 1^). The inter-quartile range of PFA ratio increase was however widely distributed, ranging from approximately 86% at the upper quartile, to a lower quartile of 28%. Other cluster groupings however saw relatively limited change in their PFA ratios, though part time farmers benefited the least in this scenario, with 19% median PFA ratio increases.

The second scenario, enhanced livestock production in the form of a 50% increase in daily milk yield, conversely had very little impact on the PFA of farm HHs in Bihar. Median net PFA ratio increase never exceeded 0.35 for any cluster (Fig. 7C). The percent change in median PFA ratios also never exceeded 10% (Fig. 7D). Small-scale crop and livestock farmers, who maintained a median of 2 heads of livestock farm^− 1^ stood to benefit the most from livestock productivity enhancing interventions, but with interquartile ranges of 4–17% PFA ratio increases. Wealthy farmers, who also maintain relatively high quantity of livestock on their farms (median 2 heads), had the second-highest median PFA ratio change at just 4%. Resource-poor agricultural laborers, conversely, did not benefit in any way (median PFA ratio change 0%), while part-time and medium-scale cereal crop farmers also benefited only marginally (1 and 2% median PFA ratio change, respectively).

The last scenario examined the effect of catastrophic drought, simulated by imposing a 90% decrease on cereals yields across clusters. The lowest median PFA ratio decrease was observed among part-time farmers, with a loss less than one (Fig. 7E). Wealthy farmers, who derived a the largest proportion of potentially consumable foods through indirect pathways, including sales of both food and cash crops, though with little reliance on livestock or off-farm income, fared worst under catastrophic drought simulations. Net median PFA decreased by two for this cluster, followed by medium-scale cereal crop farmers (− 1.3). Considering each farm HH type's percentile reduction in PFA, the catastrophic drought scenario strongly affected medium-scale cereal crop farmers (median decrease 43%). This cluster was followed by wealthy farmers reliant on food and cash crop sales, in addition to HH produced and consumed food energy (38% median PFA decrease), and by small-scale crop and livestock farmers (24% decrease), although each group showed large inter-quartile ranges (Fig. 7F). Resource-poor agricultural laborers, who depend primarily on off-farm income (often resulting from migratory labor to other States where drought may be less of a concern) to procure food, were conversely less affected.

## 4 Discussion

Using a simple indicator based on Frelat et al. (2016), we analyzed the ways in which the food security status of different farm household typologies in eastern India may be affected by changes in cropping systems and livestock management, and by environmental shocks such as drought. Typological assessment is most commonly carried out to account for farm household and livelihood heterogeneity, and to guide research and development planning processes. Different methods may be employed to collect data for typologies, the most common including household surveys and participatory approaches (Kuivanen et al., 2016, Tittonell, 2014). Farm types may be differentiated based on land holding size and market orientation (cf. Douxchamps et al., 2015, Tittonell, 2014), and/or decision-making processes (cf. Chopin et al., 2015, Bathfield et al., 2016), representing combinations of structural and functional variables.

Our results indicate that farm households in Bihar engage in widely divergent livelihood strategies that in turn affect their potential food security responses to changes in agronomic management practices, animal husbandry, and climactic shocks. Combining food security scenario analysis with farm typological assessment therefore appears to be a potentially useful tool for agricultural planners wishing to assess the impact of different development interventions on an *ex ante* basis, in order to select best-bets from different development alternatives. Although typologies and indicators represent a simplification of farm household livelihood strategies, their combined use nonetheless appears to be a useful heuristic tool to explore and assess tendencies in food security under a range of simulated circumstances. The food security ratio utilized in this paper is similar to those employed by Frelat et al. (2016), and Hammond et al. (2016) in Central America and Sub-Saharan Africa. Our study is however the first attempt to combine this indicator with typological and subsequent scenario analysis.

We first examined the potential food security implications of ‘climate smart’ agricultural practices based on the principles of conservation agriculture (CA). When well managed, CA can reduce costs and energy consumption, while at times enhancing yield when compared to farmers' conventional practices in South Asia, particularly when analyzed at the field scale (Aravindakshan et al., 2015, Gathala et al., 2015, Krupnik et al., 2014, Saharawat et al., 2010). Using research station data from Jat et al. (2014), we simulated the effect of median rice and wheat yields that reached 6.3 and 5.8 t ha^− 1^, respectively, on household food security ratios differentiated by typology. These yields are 3.78 and 4.14 t ha^− 1^ greater than the average reported rice and wheat yields for Bihar's farmers (calculated from GoB, 2016). Yet despite the increased cereal production and cost savings in our scenarios, only relatively wealthier farmers and medium-scale cereal crop farmers (constituting 50% of all farmers surveyed) stood to benefit considerably. This finding supports previous research indicating potential trade-offs and unevenness in development interventions that focus on field-scale agronomic activities without consideration of the broader socioeconomic factors that also contribute to farmers' livelihood systems (Klapwijk et al., 2014, Rosenstock et al., 2014).

Using farm survey data from Bihar, Keil et al. (2015), recently documented production advantages and adoption determinants of zero tillage (one of the elements of the CA scenario). Their results, which indicated that the adoption of zero tillage was greater among wealthy farmers with larger field sizes and a larger share of their income coming from agricultural activities, are coherent with our own. For small-scale crop and livestock farmers, CA practices may in comparison be less attractive. However even in the second scenario in which milk productivity was doubled, their PFA ratio increased only marginally. These results point to the importance of exploring development interventions that focus on improving integrated farm enterprises, rather than treating crops or livestock as separate endeavors. Aryal et al. (2014), for example, showed that the probability of incorporating maize into Bihar's cropping systems increased with livestock ownership. Mixed maize-livestock systems may therefore represent an opportunity for small-scale crop-livestock farmers, though this hypothesis requires validation.

For less endowed part-time farmers and resource-poor agricultural laborers, which comprise roughly 30% of the farmers surveyed, neither CA practices nor a boost in milk production appears to be likely to have major effects on household food security. These groups, both of which derive the majority of their income from off-farm activities, were conversely less vulnerable to simulated environmental shock in the form of drought. Households in Bihar have for some time also practiced rural out-migration as farmers seek more remunerative employment opportunities both within India and globally (Datta et al., 2014, de Haan, 2002). These practices appear to reduce the vulnerability of resource poor farmers to some extent by diversifying their income generating practices. In Bihar, the importance of off-farm activities for income generation has long been highlighted, as have diversified off-farm pursuits such as honey foraging (cf. Ashokvardhan, 2009). Similar patterns of out-migration and increased cash flows to rural households in the form of both domestic and international remittances have been observed in rural Pakistan (Kousar et al., 2016), Bangladesh (Zhang et al., 2014), and Nepal (Karki et al., 2015), indicative of the regional scale of this phenomenon.

The analytical framework here can be used to generate relatively rapid insight to assist in the targeting of interventions to different farm types and geographical regions where they are likely to be more socio-ecologically appropriate. For example, wealthier and mixed crop-livestock farmers were the largest potential beneficiaries of rice-wheat system yield increases through CA. In Nawada district, these two typologies form > 75% of survey sample, although they are < 40% in other districts. In Begusarai and Samastipur, part-time farmers and resource-poor agricultural laborers form 30–45% of our sample. Livestock is also comparatively important in these districts. Hence by combining farm typological and geographical assessment, this analysis represents a step forward compared to similar analyses that lack spatial components (e.g. Ritzema et al., 2017, Paul et al., 2016). The increasing availability of high-resolution remote sensing and georeferenced environmental data has also enabled new methods to assess the biophysical appropriateness of development interventions intended to increase farm production intensity (cf. Schulthess et al., 2015, Krupnik et al., 2017). Combining such analyses with consideration of socio-ecological variables as demonstrated in our study represents an important avenue for future research.

Our analysis relied on self-reported information with respect to food availability and income generation. Ground-truthing our study results with rapid and farmer participatory appraisals from each typological group and village would be useful to verify which development interventions may hold – or may not hold – the most promise in Bihar. Such information is particularly relevant for donors, development policy planners, NGOs and extension organizations that must choose between suites of technologies and development investment options. Likewise, our analysis used standard international coefficients for the energy contents of observed crop and livestock species, and for daily household energy requirements. These coeffieients are sufficiently robust for the PFA indicator described in this study, while having the added value of permitting cross-country comparisons. However, for detailed studies assessing the the potential effects of alternative farming or non-farming activities on within-household food security outcomes, use of locally derived coefficients (e.g. Longvah et al., 2017), and age-, gender- and physical activity profile-specific data may be more appropriate (cf. ICMR, 2010). The integration of the PFA ratio with other indicators, for example measurements of environmental externalities or ecological services provision, could also facilitate an improved understanding the primary trade-offs and synergies that may result from development interventions (Hammond et al., 2016, Paul et al., 2016, Delmotte et al., 2016). Further research should therefore consider inclusion of such multi-criteria analysis, which could be implemented alongside more advanced scenario analyses considering layered development interventions and/or by incorporating crop and livestock simulation modeling.

## 5 Conclusions

We demonstrated the use of quantitative systems analysis tools to characterize the diversity of farming systems and assess the impact of different agricultural development and environmental shock scenarios on a simple indicator of household food security. Our results showed that the impact of crop losses or the intensification of crop and livestock product production can have considerable negative or positive impact on potential food availability, respectively, although the sign of these effects will vary depending on farm households' pre-existing livelihood strategies and level of farm diversification. The effect of livestock management intensification represented by increased milk production appears to have relatively little positive effect on most farmers' potential food availability. Considering the differential impact of these scenarios among the five farm typologies observed in our dataset, we conclude that the most potentially vulnerable households are those that depend primarily on cereal crop and livestock production for their food security. Farmers who have diversified their income generation base with alternative sources of income are comparatively less vulnerable to environmental shocks in the form of drought. This research demonstrates how farm survey data can be integrated with scenario analysis to vet the potential food security responses of different development interventions or shocks by farm type. Similar scenario analysis could consider economic stresses in addition to climactic shocks or climate change scenarios, or include crop-livestock and farming system modeling, in order to better appraise and target agricultural development policy in eastern India and in other locations where smallholder agricultural systems predominate.
